# Suggestion for a new deterministic model coupled with machine learning techniques for landslide susceptibility mapping

**DOI:** 10.1038/s41598-021-86137-x

**Published:** 2021-03-23

**Authors:** Dae-Hong Min, Hyung-Koo Yoon

**Affiliations:** 1 Department of Construction and Disaster Prevention Engineering, Daejeon University, Daejeon, 300-716 Korea; 2 Department of Construction and Disaster Prevention Engineering, Daejeon University, Daejeon, 300-716 Korea

**Keywords:** Engineering, Civil engineering

## Abstract

Deterministic models have been widely applied in landslide risk assessment (LRA), but they have limitations in obtaining various geotechnical and hydraulic properties. The objective of this study is to suggest a new deterministic method based on machine learning (ML) algorithms. Eight crucial variables of LRA are selected with reference to expert opinions, and the output value is set to the safety factor derived by Mohr–Coulomb failure theory in infinite slope. Linear regression and a neural network based on ML are applied to find the best model between independent and dependent variables. To increase the reliability of linear regression and the neural network, the results of back propagation, including gradient descent, Levenberg–Marquardt (LM), and Bayesian regularization (BR) methods, are compared. An 1800-item dataset is constructed through measured data and artificial data by using a geostatistical technique, which can provide the information of an unknown area based on measured data. The results of linear regression and the neural network show that the special LM and BR back propagation methods demonstrate a high determination of coefficient. The important variables are also investigated though random forest (RF) to overcome the number of various input variables. Only four variables—shear strength, soil thickness, elastic modulus, and fine content—demonstrate a high reliability for LRA. The results show that it is possible to perform LRA with ML, and four variables are enough when it is difficult to obtain various variables.

## Introduction

The probabilistic and deterministic method has been applied to perform landslide risk assessments (LRA)^1–3^. To perform an LRA based on the probabilistic method, the probability of occurrence is determined by historical literature data and is used to define whether the determined value exceeds the reference value^4^. If there is a large amount of accumulated data linked to the criterion data, which is generally the amount of rainfall or the rainfall intensity in the same area, a reliable risk assessment can be easily performed^5^. However, it has a limitation in providing reliable results with a lack of data in the inventory to fully reflect past and present conditions when using the critical criterion of the neighborhood area^6^. The deterministic method uses geotechnical and hydraulic properties of the target area as input parameters for the LRA, and the susceptibility is generally expressed in terms of the quantitative safety factor^7^. A graphic information system (GIS) is a useful method for classifying the target area by grid, so a GIS is applied to obtain risk areas with high resolution^8^. However, the deterministic method also has limitations of time and money in obtaining various input parameters at each grid. If the measurement results are insufficient due to the circumstances, the reliability may decrease. To solve this problem, Jun et al.^9^ presented an empirically based model for an LRA, formed with the opinions of experts, and tried to improve the limitations of the deterministic method through the most important parameters of the LRA. However, this method also has limitations in quantitatively estimating LRA due to various input parameters and their different units^10^. Bui et al.^11^ constructed relationships between the soil compression coefficient and 12 input variables with different units based on a neural network. The results showed that reliable output values can be derived among various input parameters even if the units of the input values are different. Therefore, this study is focused on finding reliable constants of each parameter and proposing minimum input variables through machine learning to expand the application of the research results of Jun et al.^9^.

Various optimization methods have been used to predict susceptible areas based on the deterministic method. Chang et al.^12^ attempted landslide susceptibility mapping using a digital elevation model as a geomorphic factor, and applied logistic regression, random forest, and support vector machine. Di et al.^13^ used a gradient boosting machine to predict the susceptibility of debris flow in a watershed, and the results were compared with the outputs deduced by logistic regression, k-nearest neighbor, a support vector machine, and an artificial neural network. In recent years, researches to find a landslide susceptibility area using machine learning have been continuously conducted with selecting different algorithms for the purpose of the study. Van Dao et al.^14^, Kuradusenge et al.^15^ and Wang et al.^16^ selected the conventional algorithms including logistics regression, support vector machine, random forest, gradient boosting machine, and multilayer perceptron and however, Tien Bui et al.^17^ and Ghasemian et al.^18^ suggested to the hybrid algorithms of reduced error pruning tree algorithm and combining algorithm both stochastic gradient descent and an AdaBoost meta classifier, respectively, for improving performances. Each study also used different input variables with consideration of classifying the categories of geographical conditions and the obtained values were used for the purpose. In addition, Levenberg–Marquardt and Bayesian regularization algorithms were used as a back propagation method, which has a crucial influence on the outcome of ML^2,3,19^ in finding an optimal weight and bias for prediction^20^. Each method is developed to minimize errors when finding the inflection point through a probability technique. Therefore, in this study, ML was applied to perform an LRA based on the safety factor with input variables of various units and ranges, and the back propagation method was also applied to improve reliability.

This paper describes linear regression, a neural network, and random forest among machine learning techniques, and the back propagation methods of gradient descent, Levenberg–Marquardt, and Bayesian regularization are also explained in the background theory. The objectives for obtaining input variables are described, and the experimental results are summarized. In addition, an explanation of the interpolation method to construct the dataset based on geostatistical theory is included, and the LRA is evaluated after determining a reliable hyperparameter. The results were compared with the safety factor calculated by Mohr–Coulomb failure theory^21^ to verify the reliability.

## Background theory

### Empirical equation

Jun et al.^9^ suggested parameters closely related to the occurrence of debris flow through the analytic hierarchical process (AHP) technique to overcome the limitations of the existing deterministic method, which assumes various input parameters due to difficulty in obtaining them. The geotechnical properties were categorized into soil structure and particle distribution, the stress and strain of soil, and soil and water, considering the characterizations of solid, structural failure, and water flow. Thus, the fine content (percent passing ratio of a 0.075 mm diameter sieve)^22^, soil thickness (depth from surface to weathered bedrock)^23^, porosity (ratio between volumes of void and solid)^24^, elastic modulus (soil resistance when force is applied)^25^, shear strength (soil strength against yield or failure)^22^, hydraulic conductivity (speed of the moving fluid)^26^, saturation (volumetric water content in soil)^27^, and water content (weighted water content in soil)^28^ were selected as main parameters through an expert advisory group. The consistency ratio (CR) was used to estimate the reliability of the determined parameters, and the value was calculated as 0.0022^9^. A CR value less than 0.1 generally indicates an excellent response rate^29^. The weight factor of each parameter was determined by the relative importance and influence on debris flow. However, there is a limitation as a single formula because each factor has a different unit. Even though a scored index based on a range of values in each parameter was proposed, it was still insufficient. To improve the methodology of the previous results through machine learning, the eight selected parameters were used as independent variables, and the dependent variable was fixed to the safety factor. The safety factor was calculated using Eq. (1), derived by Mohr–Coulomb failure theory in infinite slope^21^. This study proposes a formula through the relationships among eight parameters connected to the safety factor as a true value by using machine learning:

1 $$ FS = \frac{{C_{r} + C_{s} + \cos^{2} \theta [\rho_{s} \cdot g(D - D_{w} ) + (\rho_{s} \cdot g - \rho_{w} \cdot g)D_{w} ]\tan \Phi }}{{D \cdot \rho_{s} \cdot g \cdot \sin \theta \cdot \cos \theta }} $$

where FS is the safety factor; C_r_ and C_s_ denote the cohesion (N m^–2^) of root and soil, respectively; θ and ϕ are the slope angle and friction angle (°); ρ_s_ and ρ_w_ represent soil density (kg m^–3^) and water density (kg m^–3^); D and D_w_ represent vertical soil depth (m) and water table height within soil later (m); and *g* is gravitational acceleration (m s^–2^), with a value of 9.81.

### Machine learning

Linear regression and neural network techniques, based on linear and nonlinear functions of machine learning (ML), were used to investigate the relationships between independent and dependent variables. In addition, the random forest technique based on ML was also applied to find the importance of each factor and characteristics of each technique.

#### Linear regression

**Figure 1:**
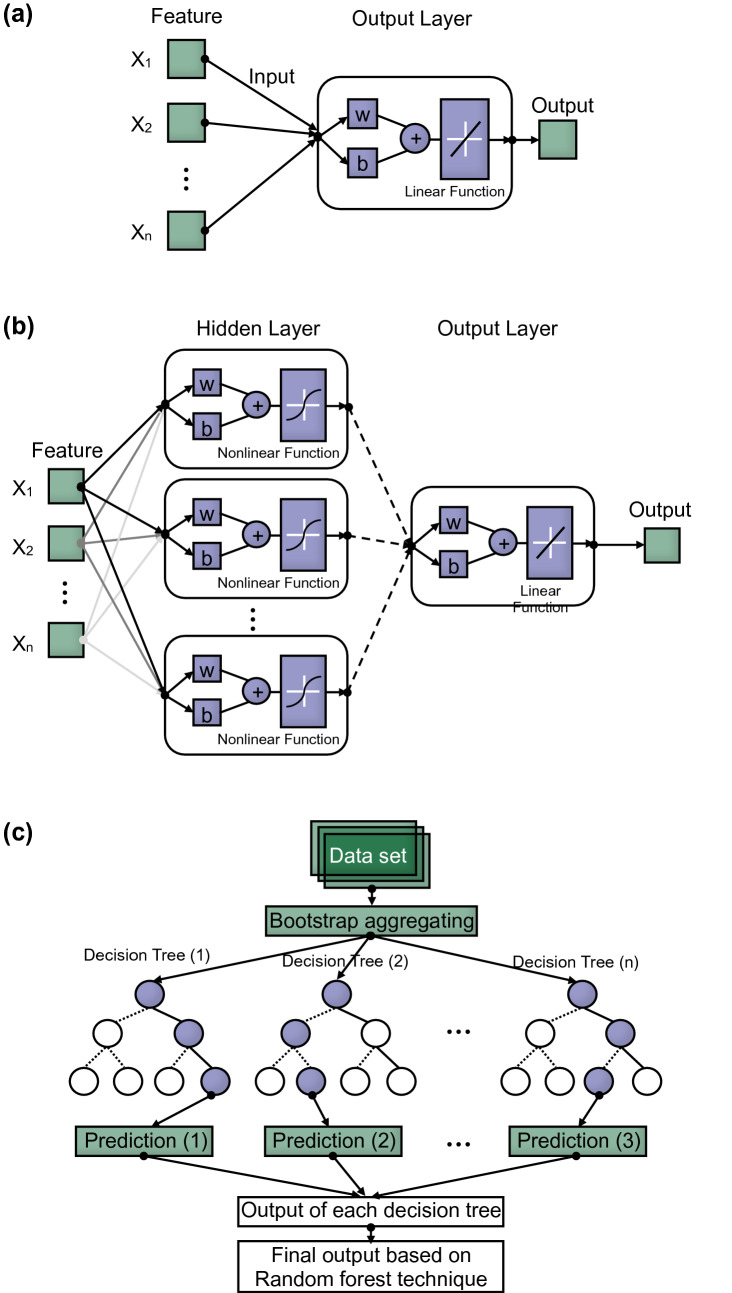
Architecture of machine learning algorithm: (**a**) linear regression; (**b**) neural network; (**c**) random forest.

Linear regression (LR) is one of the most widely used techniques in ML to find the best model suitable for the distribution of target data, and it has the advantage of identifying multidimensional linear relationships through iterative operations. The architecture and mathematical relations are addressed in Fig. 1a and Eq. (2), with weight (w), bias (b), input variable (x), and output variable based on actual outcome (H).

2 $$ H(x) \, = \, wx + b $$

#### Neural network

Neural networks (NNs) are used to solve classification and a regression of networks composed of various functions, and can understand input variables through repetitive learning, which is back propagation^30^. Back propagation updates the weight of each layer in the reverse direction by propagating the error of the output deduced by feedforward, and iterative learning is performed until the error is minimized^31^. The function to perform iterative learning is defined as an activation function, and various nonlinear functions are applied according to the characterization of the dataset. NNs are composed of input and output layers in the same way as LR, but they include a hidden layer that can perform deep learning step by step between layers, as shown in Fig. 1b. The mathematical relationship between the input and output variables is the same as Eq. (2) of LR, and the repeating process through hidden layers is different in the method of finding the weight and bias. Note that the process for finding weight and bias of LR and NN is similar based on the same optimization technique and however, they can be updated in the only NN through the hidden layer.

#### Random forest

Random Forest (RF) is an ML technique used to infer correlations between input and output variables. Thus, the technique was applied to examine the importance of eight input parameters with the safety factor. The RF architecture is composed of multiple decision trees based on an ensemble model, as shown in Fig. 1c, so the accumulation of errors occurring in each tree can be prevented. Each input parameter is randomly selected and allowed to duplicate through bootstrap aggregation, and the validation is estimated between selected data and out-of-bag (OOB) data, which are not selected data^32^. With the OOB of the original and randomly constructed tree called O_OOB_ and R_OOB_, respectively, the importance of each input factor is expressed as Eq. (3). If the calculated importance score is high, it can be judged as an important factor among each input parameter.

3 $$ {\text{Important}}\,\,{\text{score}}_{i} = \frac{{\frac{1}{t}\sum\limits_{i = 1}^{t} {\left| {O_{{OOB_{i} }} - R_{{OOB_{i} }} } \right|} }}{{\sqrt {\frac{1}{t - 1}} \sum\limits_{i = 1}^{t} {\left[ {\left( {\left| {O_{{OOB_{i} }} - R_{{OOB_{i} }} } \right|} \right) - \left( {\frac{1}{t}\sum\limits_{i = 1}^{t} {\left| {O_{{OOB_{i} }} - R_{{OOB_{i} }} } \right|} } \right)} \right]^{2} } }} $$

RF also provides the numerical relationship between two variables in input parameters, called the gray relational grade (GRG), expressed as Eq. (4):

4 $$ {\text{GRG}} = \frac{1}{n}\sum\limits_{j = 1}^{n} {\frac{{\mathop {\min }\limits_{i} \mathop {\min }\limits_{j} \left| {X_{R,J} - X_{C,J} } \right| + \delta \mathop {\max }\limits_{i} \mathop {\max }\limits_{j} \left| {X_{R,J} - X_{C,J} } \right|}}{{\left| {X_{R,J} - X_{C,J} } \right| + \delta \mathop {\max }\limits_{i} \mathop {\max }\limits_{j} \left| {X_{R,J} - X_{C,J} } \right|}}} $$

where X_R_ and X_C_ are referencing and comparing variables, j is a sequence of each variable, and δ is the resolving coefficient, generally assumed to be 0.5^33^. If the calculated value is close to + 1 or − 1, the relationship shows a high correlation.

#### Cost function

The cost function indicates the performance of ML and is calculated as the square of the difference between the predicted value (H), calculated by weight (w) and bias (b), and the actual value (Y), as shown in Eq. (5). The cost function shows a quadratic parabolic distribution according to w and b. The point at which the slope of the parabola is at a minimum shows the best performance, so the partial derivative is performed at each point of the parabola, as shown in Eq. (6).

5 $$ \cos t(w, b) = \frac{1}{m}\sum\limits_{i = 1}^{m} {(H(x_{i} ) - Y_{i} )^{2} } $$

6 $$ w^{\prime},b^{\prime} = w,b - \eta \frac{\partial }{\partial w,\partial b}\cos t(w,b) = w,b - \eta \frac{{\partial J_{n} }}{{\partial a_{s}^{(L + 1)} }} \cdot \frac{{\partial a_{s}^{(L + 1)} }}{{\partial z_{s}^{(L + 1)} }} \cdot \frac{{\partial z_{s}^{(L + 1)} }}{{\partial w_{s,r}^{(L)} ,\partial b_{s,r}^{(L)} }} $$

where w′ and b′ indicate the weight and bias from which the error has been removed; and η is the learning rate, which is the degree of the learning step as a partial differential interval and is generally 0.001^34^. The cost function can be subdivided into weight and bias at corresponding nodes through the chain rule. L denotes the number of the hidden layer, and s and r indicate the node numbers of and before the L layer. Z_s_ is the sum of weights and biases corresponding to each node, and a_s_ is the value of the activation function that is applied to Z_s_. J_n_ is the Jacobian matrix of the error computed at the hidden layer. A method of using partial differentiation at a certain learning rate is called gradient descent, and this is a commonly used method to find w and b in ML. However, this method has a limitation, the reliability of the cost function depends on the learning rate interval. Methods to overcome this have been proposed. In this study, Levenberg–Marquardt and Bayesian regularization algorithms were additionally considered to improve reliability.

##### Levenberg–Marquardt algorithm

The Levenberg–Marquardt (LM) algorithm is suitable for solving nonlinear least square problems. Marquardt developed the algorithm in 1963 based on a theory suggested by Levenberg in 1944. This method estimates an optimal w and b based on the Gauss–Newton method and can provide the solution by locally approximating the nonlinear function as a linear function by generalizing the multivariate vector. LM can flexibly change the learning rate without fixing a value. A small interval of the learning rate is given when the solution is stably converged, and a large interval value is set in the opposite case. Therefore, it is an advantage to compensate for a divergent differential value due to a fixed learning rate in gradient descent. The slope of the cost function based on LM (slope_LM_) is calculated by the Jacobian matrix (J), as shown in Eq. (7). diag (JrTJr), which is an intrinsic value of the Hessian matrix, represents the curvature of the function and is used to determine the magnitude of the learning rate^35^:

7 $$ slope_{LM} = (J_{{}}^{T} J + \eta diag(J_{{}}^{T} J))^{ - 1} J_{{}}^{T} r(p) $$

where r(p) is the error of the model, and diag is the diagonal matrix.

##### Bayesian regularization (BR)

Bayesian regularization (BR) is a method proposed by MacKay^36^ that uses a Hessian matrix in the same way as the LM algorithm. However, the weight is determined according to a random variable by adding Bayesian theory^37^. BR is mathematically expressed as a probability density function with weight (w), dataset (D), function parameters (α, β), and a particular model (M), as shown in Eq. (8):

8 $$ P(w\left| {D,\alpha ,\beta ,M)} \right. = \frac{{P(D\left| {w,\beta ,M)P(w\left| {\alpha ,M)} \right.} \right.}}{{P(D\left| {\alpha ,\beta ,M)} \right.}} = \frac{{\exp ( - (\beta E_{D} + \alpha \sum\limits_{K = 1}^{l} {} \sum\limits_{i,j = 1}^{m} {(w_{ij}^{k} } )^{2} )}}{{(\int {\exp ( - \alpha \sum\limits_{K = 1}^{l} {} \sum\limits_{i,j = 1}^{m} {(w_{ij}^{k} } )^{2} )dw} )(\frac{2\pi }{\beta })^{\frac{N}{2}} }} $$

where P(D∣w, β, M) is the possibility that the weights, assumed by the likelihood function, can appear, and P(w∣α, M) shows the prior density representing the weight of the previous step. P(D∣α, β, M) is a normalization factor; the total probability of occurrence can be finalized to 1. Each probability can be extensively expressed with a sum of squared error (E_D_) through Gaussian noise and weight based on the maximum posterior probability deduced to the optimal value.

### Performance

Studies based on the ML technique generally use the root mean square error (RMSE) and mean square error (MSE) to find the performance-comparing quantitative value^38–41^. In this method, RMSE and MSE were also used to verify the reliability of each optimization method. The mathematical expressions of RMSE and MSE are as follows:

9 $$ RMSE = \sqrt {\frac{1}{n}\sum\limits_{i = 1}^{n} {(Z(s_{i} ) - \hat{Z}(s_{i} ))^{2} } } $$

10 $$ MSE = \frac{1}{n}\sum\limits_{i = 1}^{n} {\left| {Z(s_{i} ) - \hat{Z}(s_{i} )} \right|} $$

where $Z(s_{i} )$ and $\hat{Z}(s_{i} )$ denote actual and calculated safety factors, and n is the amount of data.

### Geostatistical method

The kriging method is used to obtain the value at an unknown point considering spatial variability through an interpolation technique and has been widely adopted for its advantage of reducing error variance^42^. The error variance (σ(P_0_)) at point (P_0_) is defined by Eq. (11) with semi-variance (γ(P_n_,P_0_)) between the measured value at point (P_0_) and the predicted value at point (P_n_), and Lagrange multiplier (ψ (P_0_)).

11 $$ \sigma^{2} (P_{0} ) = \sum\limits_{n = 1}^{n} {\lambda_{n} \gamma (P_{n} ,P_{0} ) + \psi (P_{0} )} $$

where λ_n_ denotes the weighting factor.

The variogram, which can reflect the variance of λ_n_ between P_0_ and P_n_, was defined with a nugget, sill, and range to minimize the error. The nugget and sill are the non-zero variogram and maximum amplitude, respectively. The range is the distance between the variogram and the sill.

## Methodology

### Site description

**Figure 2:**
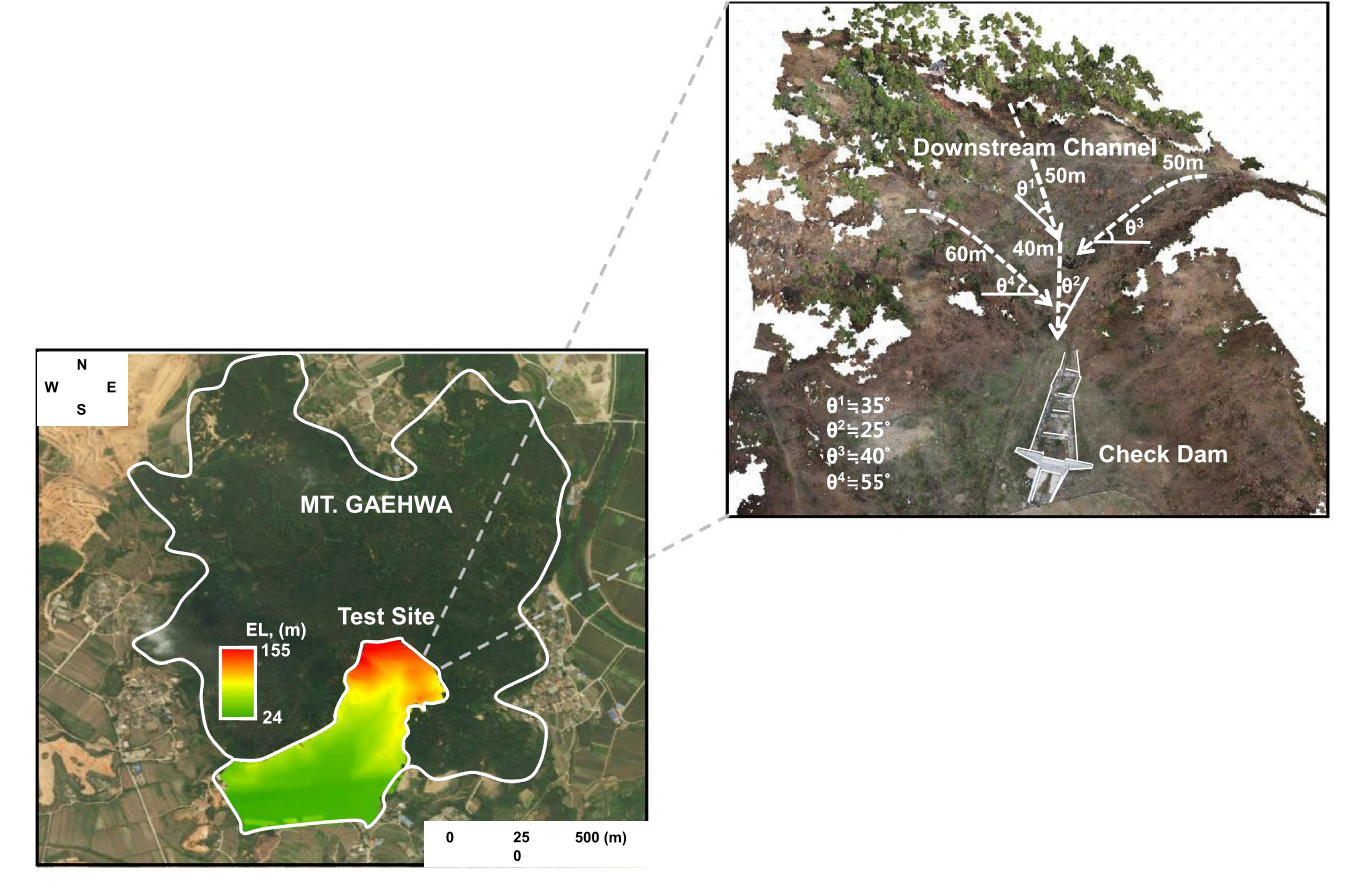
Site description. The picture of left side was captured through https://map.kakao.com and the detailed topography, placed on right side, was directly obtained by on-site geological survey with UAV survey. The agisoft viewer was applied to find the topography based on measured digital elevation model.

Gaehwa Mountain, located in the city of Sejong, South Korea, at 36° 29′ 10.77″ N–36°29′07.65″ ′′ N latitude and 127°18′46.03″ E–127°18′47.73″ E longitude, was selected as the experimental site because debris flow occurred in this area about five years ago. The whole view of this slope is shown in Fig. 2, and the range of the digital elevation model, derived by aerial surveying, is approximately 24–155 m. The study area is composed of a main stream 90 m in length and two branch streams 60 and 50 m in length, which are connected downstream of the main stream on the left and right. The slope of the main stream is about 25–35°, and the slope of the branch streams is relatively high, about 40–55°. In addition, a large amount of soil was deposited downstream due to the debris flow, and a check dam had already been installed to prevent disasters.

### Experiment

**Figure 3:**
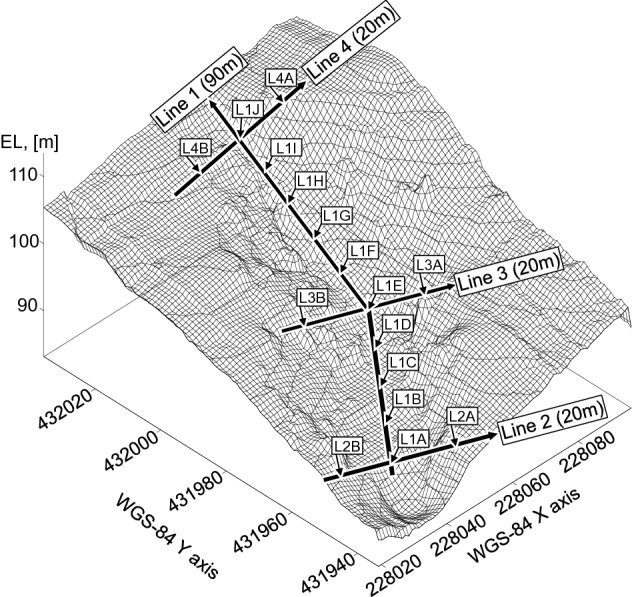
Experimental profiles. Lines 1, 2, 3, and 4 denote seismic and resistivity survey profiles. Arrows show points for performing penetration tests and extracting soil; arrow interval is 10 m. The only grid was built from software of Surfer. WGS: World Geodetic System.

Field and laboratory experiments were performed to obtain the input parameters of the empirical equation. Field experiments were conducted with a seismic survey, an electrical resistivity survey, a dynamic cone penetration test (DCPT), and time domain reflectometry (TDR). The disturbed samples were also extracted to perform sieve analysis in the laboratory. The seismic and electrical resistivity surveys used four profiles, including main and branch streams, as shown in Fig. 3. The geophone spacing for the seismic survey was set to 2 m, and the commonly used drop hammer was used as the source. Electrodes for measuring electrical resistivity were installed at the same intervals as the seismic survey, and the Wenner array method was applied. The detailed contents of seismic and electrical resistivity surveys were replaced with a reference^43^. DCPT and TDR were performed at about 10 m intervals at the same locations at which the geophysical survey was conducted, and 16 in situ tests were performed. The principle and experimental procedure of the DCPT and TDR are based on the following papers^44–46^. In addition, soil sampling was carried out in the same area as the in situ exploration. The sieve analysis was performed with a procedure suggested by ASTM^47^. The field test and sampling location based on world geodetic system (WGS) coordinates are shown in Fig. 3.

## Results

### Field and laboratory test results

**Figure 4:**
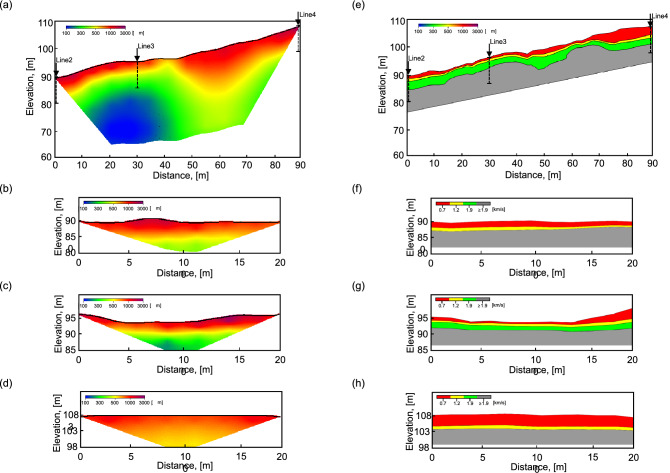
Geophysical survey results: (**a**) line 1; (**b**) line 2; (**c**) line 3; (**d**) line 4 for electrical resistivity (**e**) line 1; (**f**) line 2; (**g**) line 3; (**h**) line 4 for elastic wave.

A cross-sectional diagram of resistivity is shown in Fig. 4a–d, and the relatively high range (over ≈1000 Ωm) is distributed from the surface to a depth of about 10–15 m. High electrical resistivity was strongly demonstrated in the upper area of the slope (around Line 4) due to the high altitude. In the lower part of the slope, high amounts of groundwater can be collected due to the geological characteristics of the deep valley, as shown in Figs. 2 and 3. Therefore, a low electrical resistivity of about 100 Ωm appeared at a depth of 10 m at a slope distance of 20–40 m. The electrode spacing of lines 2, 3, and 4 was the same as that of line 1, but the surface depth was relatively less than that of line 1 due to the shorter distance used for performing the experiment. The results of lines 2, 3, and 4 are similar to those of line 1, with a high electrical resistivity of around 1000 Ωm on the surface. Low electrical resistivity, which was similar to line 1 at an 85 and 90 m depth, was deduced at a distance of 5–15 m of lines 2 and 3. The results of electrical resistivity were used to convert porosity and hydraulic conductivity into input parameters in the empirical equation. Archie’s law was used to obtain porosity^48,49^, and the cementation factor (m) and tortuosity factor (α) were set to 1.3 and 1 with reference to previous studies^50,51^. Hydraulic conductivity was deduced by the Kozeny-Carman equation through porosity, derived by Archie’s law, and the effective diameter, derived by sieve analysis^46^.

The result of seismic wave velocity is shown in Fig. 4e–h. The strata were divided into a colluvium layer, 0.7 km/s, sediment layer, 1.2 km/s; sedimentary rock layer, 1.9 km/s; and a soft rock layer, 1.9 km/s or more, based on the literature^52–54^. The colluvium layer deposited on the surface was the most affected stratum by debris flow, and the thickness was estimated at 0.3–3 m in lines 1, 2, 3, and 4. Even though it is difficult to systematically compare electrical resistivity and seismic wave results, since the properties that affect each technique are different, the upper (0–10 m) and lower (70–90 m) distances of the slope were thick, and the middle distance (20–40 m) was thin at Line 1 with similar electrical resistivity. The thickness of the surface at lines 2, 3, and 4 also shows a similar trend to the that of areas with high electrical resistivity ranges. The distribution of elastic wave velocity at each location was used to calculate the elastic modulus as an input parameter in the empirical equation^55–58^.

**Figure 5:**
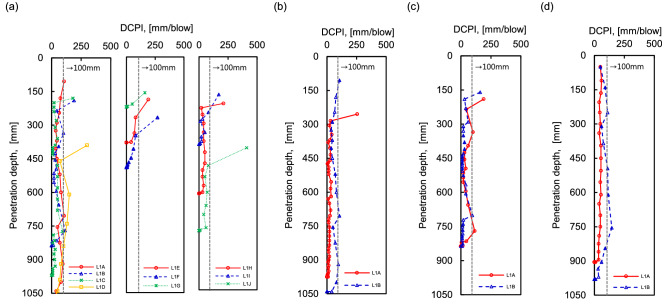
DCPT results: (**a**) line 1; (**b**) line 2; (**c**) line 3; (**d**) line 4.

The penetration depth of the DCPT at each line was plotted as shown in Fig. 5, and the results of line 1 were divided into lower (L1A to L1D), middle (L1E to L1G), and upper (L1H to L1J) parts of the slope. The initial penetration depth was more than 100 mm in the lower, middle, and upper parts in Line 1, and lines 2 and 3 also show the same results. Note that a large amount of soil was moved from the upper part to the ground due to debris flow, so a large penetration depth was recorded at initial impaction. Therefore, it is assumed that the surface is weak. The initial penetration depth of line 4 was about 50 mm on average, which is smaller than the depth obtained by other lines, indicating relatively stiff ground. The reason for this is that the soil of the initial zone for the occurrence of debris flow, in the upper area, had already moved to the lower part, so the strength of the surface is high. The DCPT test results were used to estimate soil thickness as an input variable, and the experiment was performed until the penetration depth was ≈0 mm^44,45,59^. The final penetration depth was recorded as approximately 700, 1000, 800, and 950 mm on average for lines 1, 2, 3, and 4, respectively. The shear strength in the empirical equation was also estimated through the results of the DCPT, and the correlation equation, which was calculated in similar geological conditions of weathered soil on the slope, was applied^60^.

**Figure 6:**
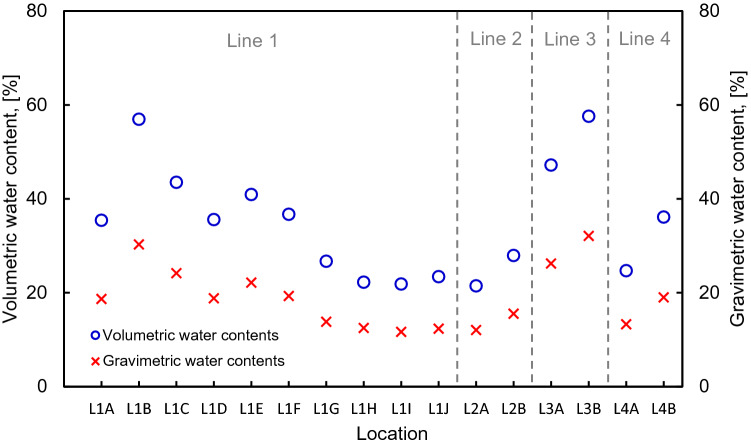
Distributions of volumetric and gravimetric water contents.

Figure 6 shows the volumetric water content measured by TDR and the gravimetric water content derived from laboratory experiments through extracted samples, which were applied to the input parameters in the empirical equation. L1A-L1F, L3A, and L3B, which are catchment basins formed by steep slopes, showed relatively high water content,the ranges of volumetric and gravimetric water content were 35–57% and 18–32%. For the opposite reason, the ranges of volumetric and gravimetric water content were found to be relatively low at 21–36% and 11–19% in L1G-L1J, L4A, and L4B, which are the upper areas of the slope. The volumetric water content was higher than the gravimetric water content under a three-phase soil system, and the difference was mainly due to the soil bulk density, assuming that the water density is uniform. The difference between volumetric and gravimetric water content was large in the catchment areas at L1A-L1F, L3A, and L3B (approximately 1.53 g cm^–3^ on average). The reason for this difference is that the weight per unit volume was increased due to the mixed fine particles from movement into the lower part of the slope.

**Figure 7:**
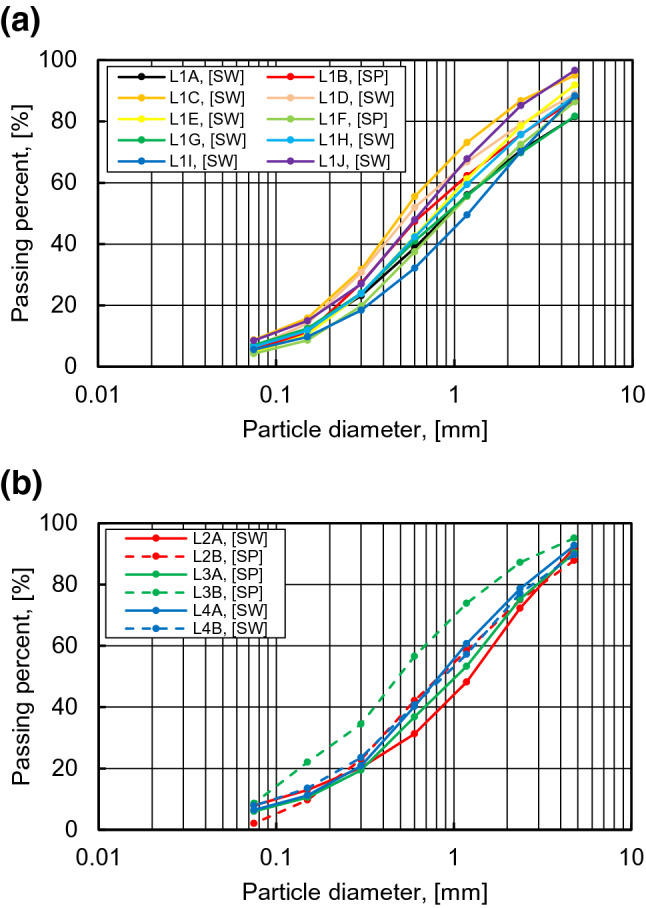
Grain-size distribution curves: (**a**) line 1; (**b**) line 2, 3 and 4.

Figure 7 shows the results of sieve analysis, which were deduced from well-graded soil (SW), based on the unified soil classification system (USCS). The fine content was estimated as the amount that passed through a #200 sieve,the catchment areas of L1A, L1C, and L2A and the initial area of L4B showed high values of 7.8%, 8.7%, 7.5%, and 7.7%, respectively. The fine content in this area was calculated as 6.3% on average, as shown in Table 1. The coefficients of uniformity (Cu) and curvature (Cc) are also shown in Table 1. All values show that Cu is greater than 4 and Cc is distributed in the range of 1–3 as a well-graded particle.

**Table 1:** Detailed sieve test results.

Location		Fine content (%)	Coefficient of uniformity (C_u_)	Coefficient of curvature (C_c_)
Line 1	L1A	7.8	15.0	1.1
	L1B	5.8	8.5	0.7
	L1C	8.7	8.2	1.3
	L1D	6.1	8.5	1.1
	L1E	4.4	7.3	0.9
	L1F	4.1	9.4	0.8
	L1G	6.9	14.3	1.0
	L1H	6.3	10.9	1.2
	L1I	5.3	12.0	1.1
	L1J	8.3	10.0	1.5
Line 2	L2A	7.5	17.8	2.2
	L2B	1.8	8.0	0.8
Line 3	L3A	5.8	10.0	0.9
	L3B	7.3	8.8	0.9
Line 4	L4A	6.7	8.0	1.1
	L4B	7.7	15.0	1.1

Finally, the eight input parameters in the empirical equation were derived through the previous experiments. Porosity and hydraulic conductivity were obtained by converting electrical resistivity, and elastic modulus was derived from elastic wave velocity. DCPI was used to estimate shear strength and soil thickness, and saturation was used as the volumetric water content converted to TDR. In addition, gravimetric water content and fine content were obtained through laboratory tests.

### Dataset

**Figure 8:**
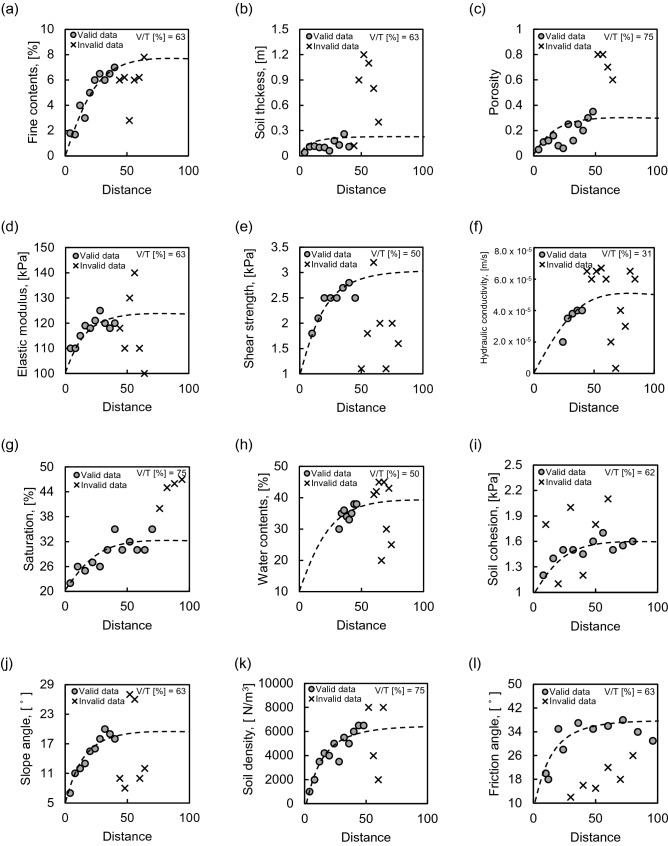
Variograms of input variables: (**a**) fine content; (**b**) soil thickness; (**c**) porosity; (**d**) elastic modulus; (**e**) shear strength; (**f**) hydraulic conductivity; (**g**) saturation; (**h**) water content; (**i**) soil cohesion; (**j**) slope angle; (**k**) soil density; (**l**) friction angle.

**Figure 9:**
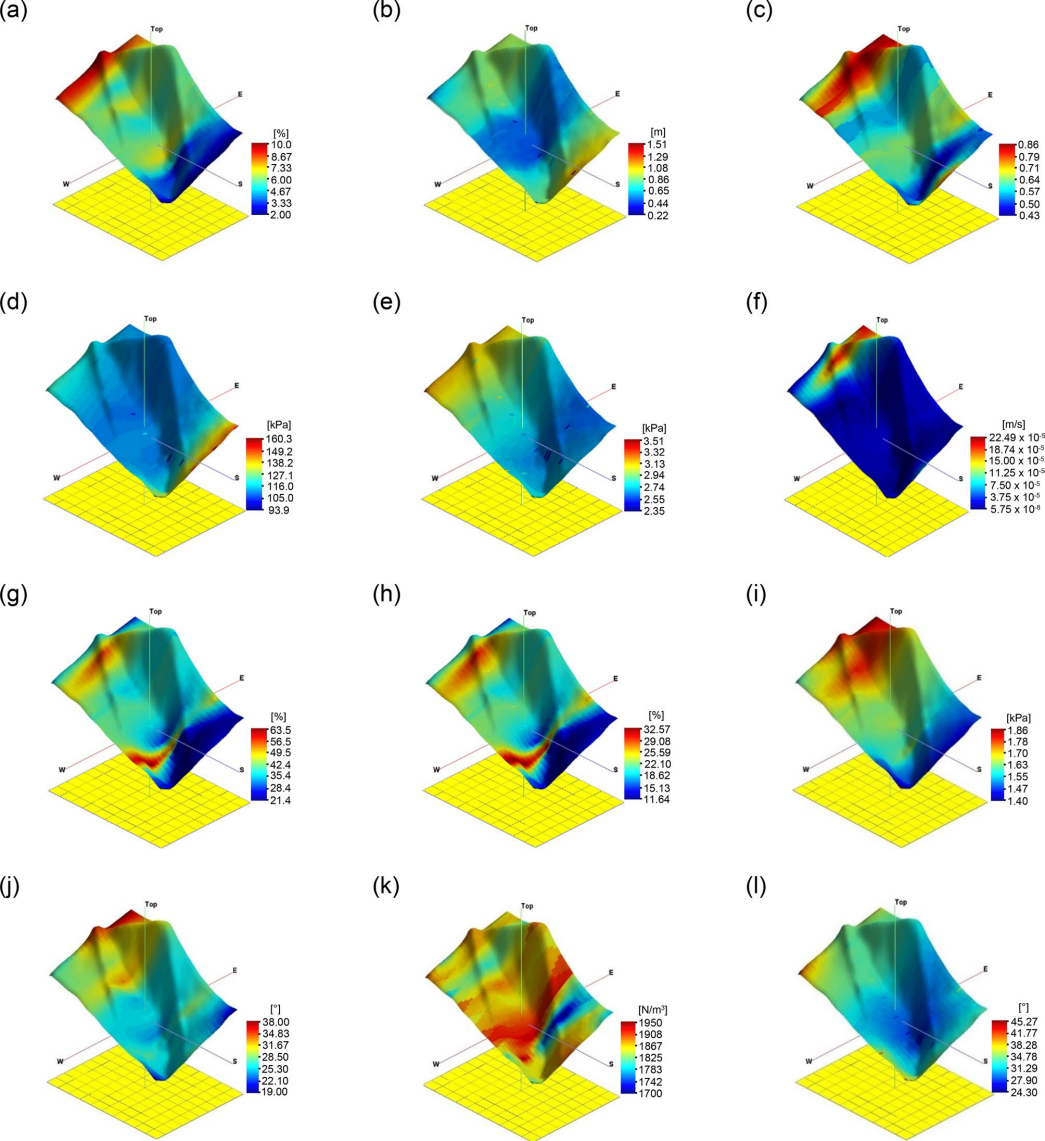
Kriging results of input variables: (**a**) fine content; (**b**) soil thickness; (**c**) porosity; (**d**) elastic modulus; (**e**) shear strength; (**f**) hydraulic conductivity; (**g**) saturation; (**h**) water content; (**i**) soil cohesion; (**j**) slope angle; (**k**) soli density; (**l**) friction angle. The software of SGeMS beta (v.2.5b) was applied to draw this figure.

The objective area was divided into 1800 grid cells of 1 m each to examine the relationship between dependent and independent variables considering spatial variability. The variables were set to the eight factors of the empirical equation and safety factor to perform deep learning, including LR, an NN, and an RF. Datasets of dependent and independent variables were constructed at the experimental grids. The dependent variable was calculated with Eq. (1) and soil cohesion, slope angle, soil density, and friction angle were determined referring to previous results in the same area^61^. The vertical water table height and root cohesion were fixed at 0.5 m and 100 N m^–2^, respectively, the same as the values ​​used in the previous study^61^. For soil depth, the same value as the empirical equation based on the field experiment was applied. However, the field penetration and laboratory tests were conducted on only 16 positions,thus, more data were required to perform LR, an NN, and an RF. Therefore, the geostatistical technique, a specialized interpolation method with kriging, was applied to determine the dependent and independent variables of the remaining grids^62^. The sill, nugget, and range values were derived from the distribution of each dataset based on geostatistics, and the constant values are shown in Table 2. The Stanford Geostatistical Modeling Software (SGeMS) beta (V2.5b), which is an open-source computer package for solving spatially related variables, was used to perform kriging analysis. The resolution of a variogram consisting of a sill, nugget, and range is known to be superior, as the nugget-to-sill ratio is smaller^63^. Among exponential, spherical, and Gaussian models, spherical and Gaussian models with the smallest nugget-to-sill ratio were selected. Note that the spatial distributions of the measurement result well correlated with the spherical and Gaussian models. Figure 8 shows 12 variograms, including 8 and 4 input parameters for the empirical equation and Eq. (1), respectively. Among the 16 tests, the data used for kriging in accord with the variogram were expressed as valid data, and the rest were indicated as invalid data. The valid data to total data ratio was calculated to be about 31–75%. Porosity, saturation, and soil density showed the highest ratio, while hydraulic conductivity showed the lowest ratio. The reason for this is that the spatial variability in this area is different for each parameter. The 3D kriging result based on the derived variogram is shown in Fig. 9, showing the distribution of each parameter. Finally, 1800 datasets including artificial data based on the geostatistical technique were constructed.

**Table 2:** Factors of variogram in each input variable.

	Nugget effect	Sill	Type	Range
Fine content	0	2	Spherical	16
Soil thickness	0	0.1	Spherical	9.6
Porosity	0	0.01	Spherical	12.8
Elastic modulus	0.0001	0.0002	Spherical	80
Shear strength	30,000	120,000	Gaussian	92
Hydraulic conductivity	0	5.5E−09	Spherical	80
Saturation	0	300	Spherical	130
Water content	0	80	Spherical	132
Soil cohesion	0	22,000	Spherical	75
Slope angle	0	20	Spherical	38
Soli density	0	4000	Spherical	12
Friction angle	8	30	Spherical	50

### Machine learning results

#### Linear regression

**Figure 10:**
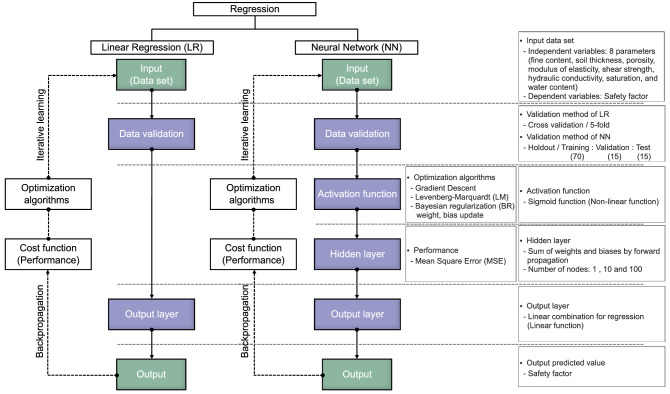
Flow chart of linear regression and the neural network.

**Figure 11:**
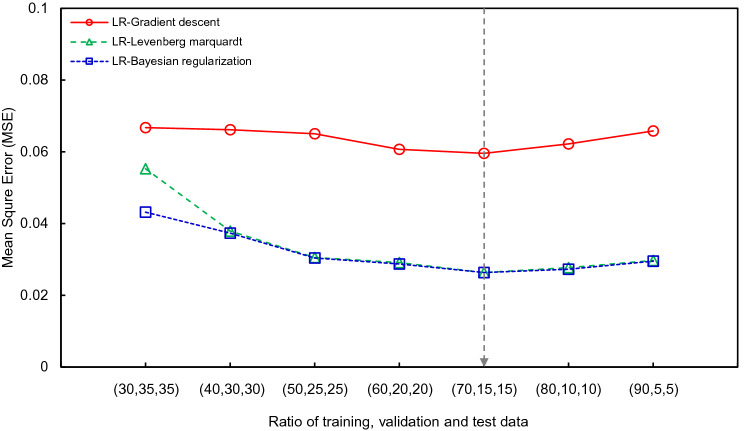
MSE according to selected ratio in linear regression.

**Figure 12:**
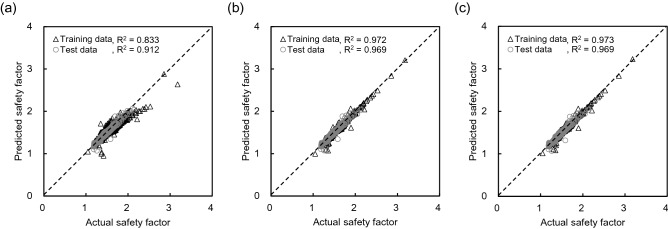
Comparison between the predicted and actual safety factors based on linear regression: (**a**) gradient descent algorithm; (**b**) Levenberg–Marquardt algorithm; (**c**) Bayesian regularization algorithm.

**Figure 13:**
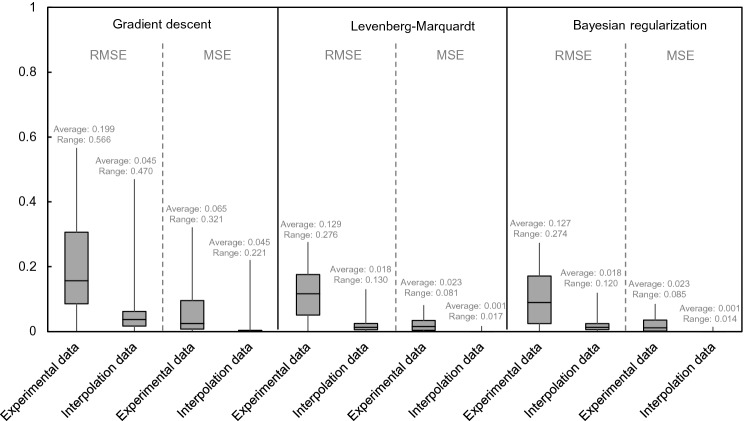
Distribution of root mean square error (RMSE) and mean square error (MSE) based on linear regression.

The multi-LR model was applied to find the relationship between independent and dependent variables with gradient descent, Levenberg–Marquardt (LM), and Bayesian regularization (BR). The flow chart of performing linear regression was expressed in Fig. 10. The ratio of training and test data was determined to be 7:3 to perform cross-validation based on the hold-out validation technique,thus, 1260 and 540 pieces of data were randomly used for training and test procedures, respectively. The MSE value according to the validation ratio is shown in Fig. 11, and the 7:3 ratio showed the smallest value. The learning rate and epoch were set to 0.001 and 1000, respectively. The comparison between the actual and predicted safety factor based on LR is addressed in Fig. 12. In the gradient descent algorithm, the relationship showed almost linear behavior when it was lower than ≈1.8, but the safety factor was underestimated at over ≈1.8. The actual safety factor showed a linear relationship with the predicted safety factor when using LM and BR algorithms. Therefore, the coefficient of determination (R^2^) was 0.96 for training and test data in LM and BR. Although BR has a slightly higher R^2^ than LM, they both provide more reliable results than the gradient descent algorithm. In addition, root mean square error (RMSE) and mean square error (MSE) were calculated through Eqs. (9) and (10) to verify the reliability of the artificial dataset dividing experimental and interpolation data, and the results are shown in Fig. 13. The average RMSE and MSE values based on interpolation were 13–22% and 4–69% smaller, respectively, than those based on the experimental data in all algorithms. This result shows that the reliability of 1800 pieces of data, constructed by the interpolation method, is high and there are no outlier data. In addition, BR shows lower RMSE and MSE ​​than any other algorithm with the same trend of R^2^. Note that BR is the most reliable algorithm,the relationship between dependent and independent variables with BR based on the multi-LR model is shown in Eq. (12):

12 $$ Y = 13.9362 + 0.3744 \cdot X_{1} - 0.9758 \cdot X_{2} + 6.3219 \cdot X_{3} - 0.1350 \cdot X_{4} - 5.0673 \cdot X_{5} - 0.003072 \cdot X_{6} + 1.5053 \cdot X_{7} - 3.0019 \cdot X_{8} $$

where X_1_, X_2_, X_3_, X_4_, X_5_, X_6_, X_7_, and X_8_ denote the fine content, soil thickness, porosity, the modulus of elasticity, shear strength, hydraulic conductivity, saturation, and moisture content as independent variables. Y represents the safety factor as the dependent variable.

#### Neural network

**Figure 14:**
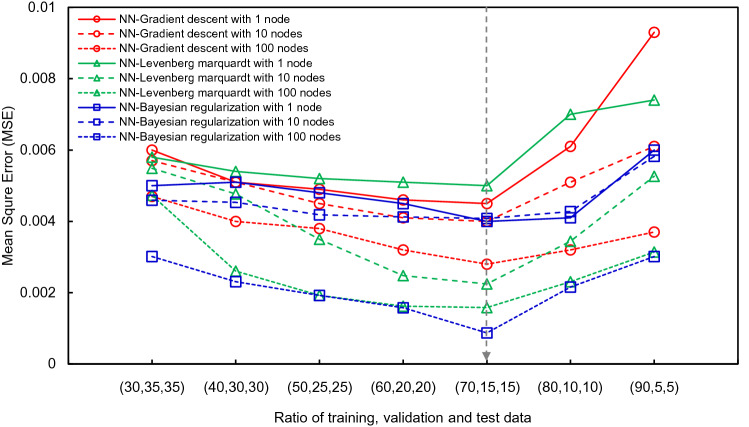
MSE according to selected ratio in neural network.

The eight input factors and safety factors were designated as independent and dependent variables, respectively, as multi-LR, and the relationships between two variables were then investigated by applying a neural network. The procedures of regression based on an NN was plotted in Fig. 10. The NN focused on the nonlinear relationships between variables, and a sigmoid function was applied. The ratio between training and test data was 7:3 as multi-LR, and the reliability of prediction was improved with cross-validation. The MSE value according to the validation ratio in the NN is shown in Fig. 14. The learning rate and the number of learning iterations were set to 0.001 and 1000, respectively. The number of nodes and the optimization method were changed as hyperparameters, and the number of nodes was divided into 1, 10, and 100. The gradient descent, LM, and BR of the optimization method were selected in the same way as LR.

**Figure 15:**
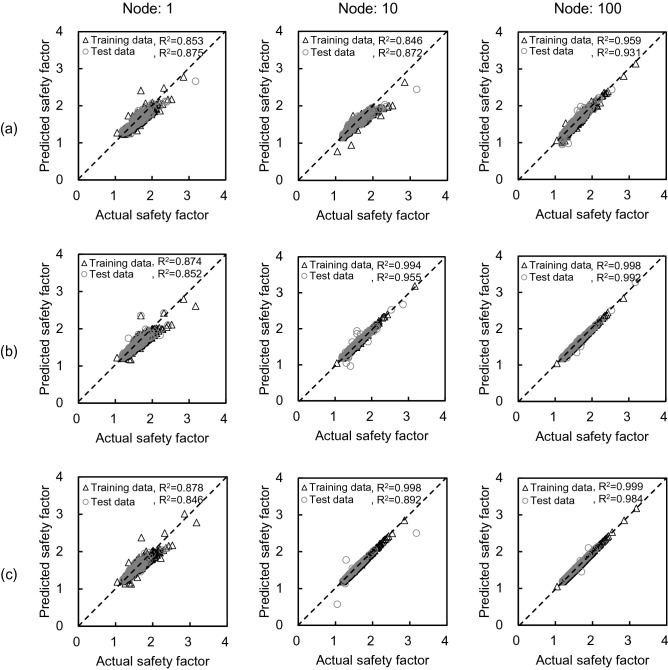
Comparison between predicted and actual safety factors based on neural network: (**a**) gradient descent algorithm; (**b**) Levenberg–Marquardt algorithm; (**c**) Bayesian regularization algorithm.

**Figure 16:**
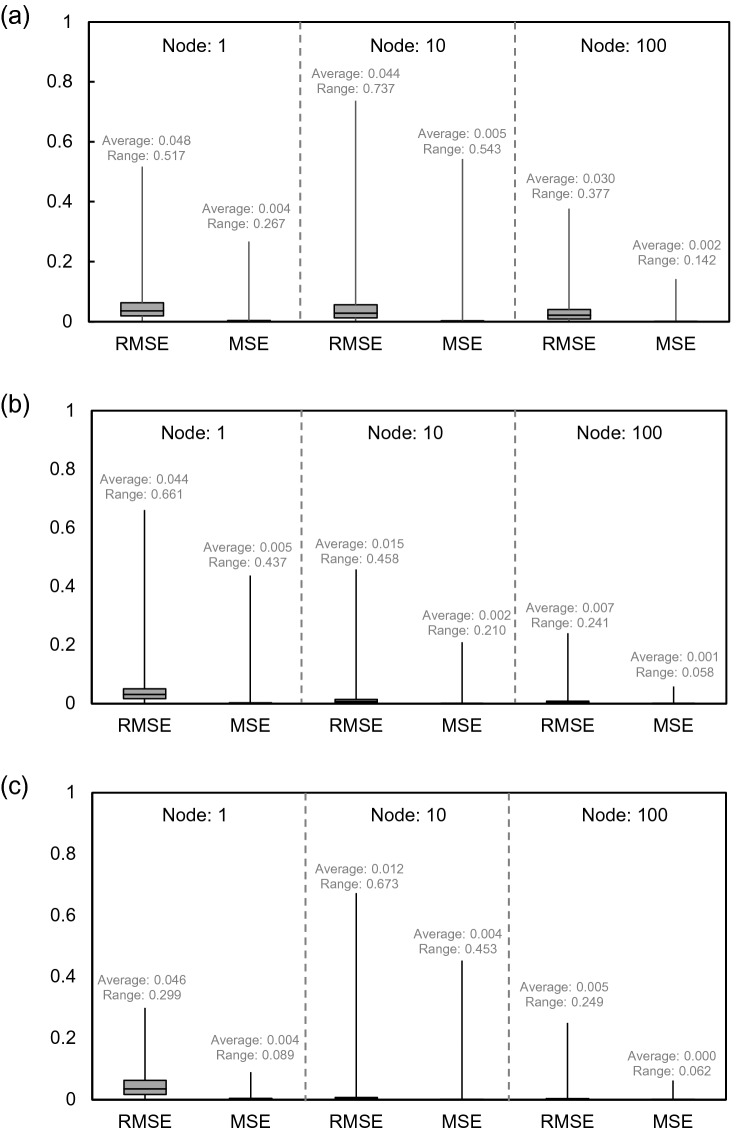
Distribution of RMSE and MSE based on neural network: (**a**) gradient descent; (**b**) Levenberg–Marquardt; (**c**) Bayesian regularization.

The relationship between actual and predicted safety factors is compared in Fig. 15 and was classified with the optimization method and the number of nodes. As the number of nodes increases, R^2^ increases in all optimization methods and the linearity is excellent. When the number of nodes is 100, R^2^ is 0.945, 0.995, and 0.992, on average, in gradient descent, LM, and BR, respectively. In addition, the R^2^ of LM and BR was larger than the R^2^ of gradient descent, similar to the result of multi-LR. With the NN, LM showed excellent performance with a slight difference. RMSE and MSE values of each optimization method according to the number of nodes are shown in Fig. 16. Similar to the R^2^ result, the calculated value tends to decrease as the number of nodes increases, and LM and BR show relatively smaller values than gradient descent. In addition, LM has a smaller range than BR when there are 100 nodes. Therefore, LM was selected as the best optimization method, and the number of nodes was fixed to 100 to operate the NN.

#### Random forest

**Figure 17:**
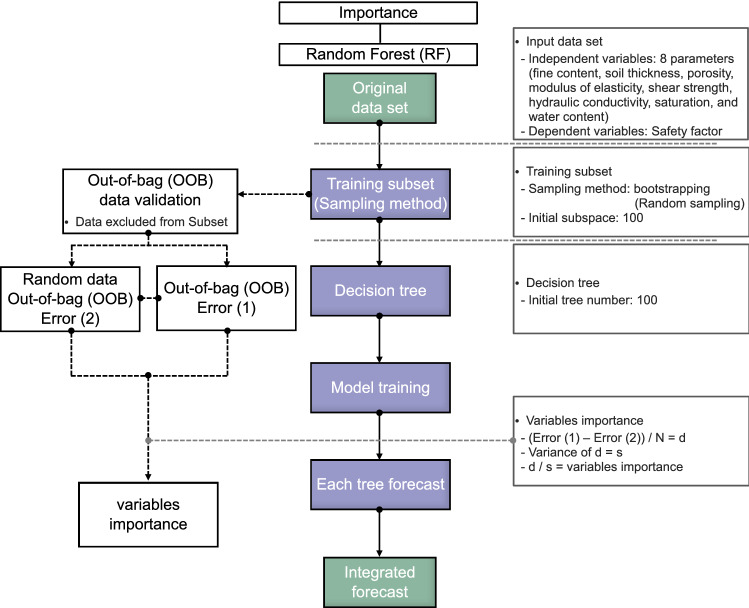
Flow chart of the random forest.

**Figure 18:**
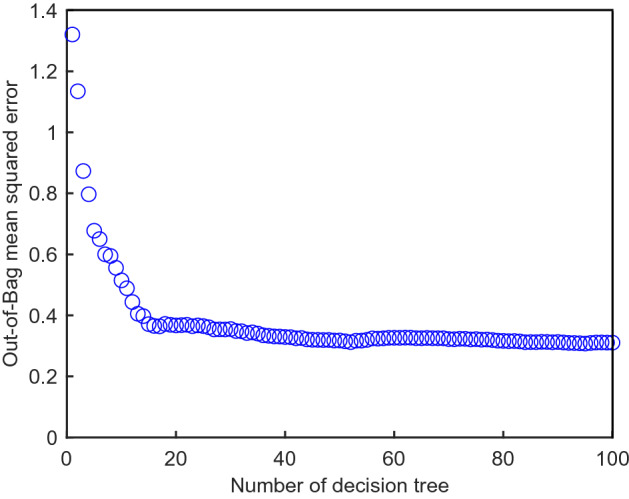
Out-of-bag refers to the squared error according to the number of trees.

**Figure 19:**
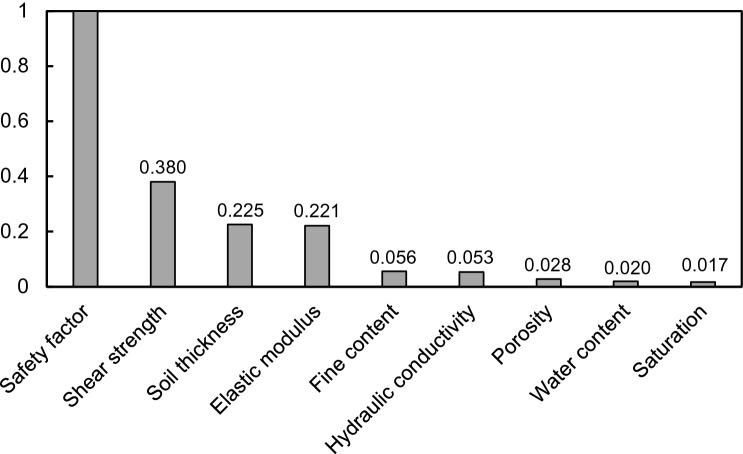
Importance of input variables based on random forest.

**Figure 20:**
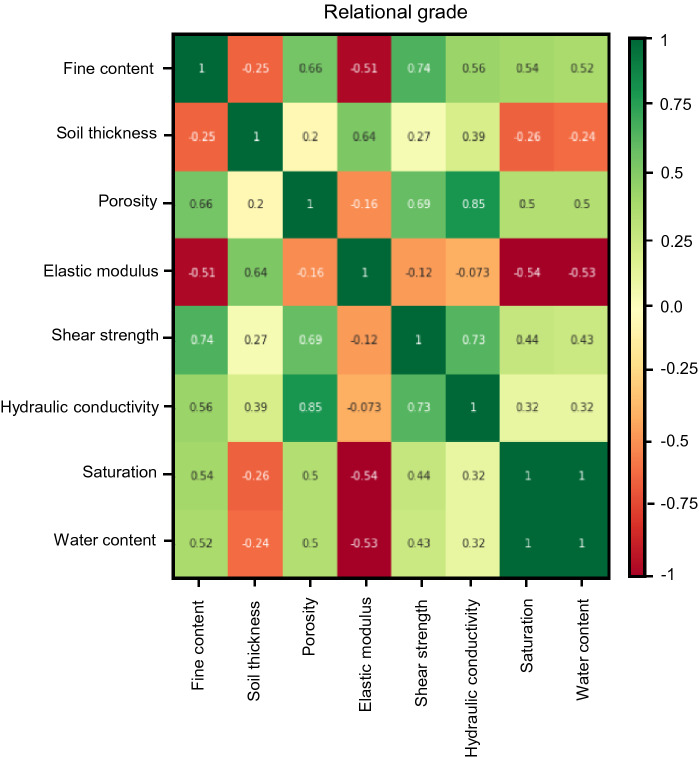
Gray relational grade (GRG) results based on random forest.

The random forest (RF) algorithm was applied to estimate the importance of the eight independent variables. The random variable (m) was determined as 3, referring to a previous study selecting at least one-third of the independent variables^64^. The flowchart of RF is summarized in Fig. 17 and the number of decision trees was considered by calculating the OOB error according to the number of trees, as shown in Fig. 18. The error (shown in Fig. 18 was found to have converged from about 20 decision trees or more, so the decision tree was set to 20. The importance of each factor was calculated as a score for predicting the safety factor through Eq. (3), and the result is shown in Fig. 19. Based on the quantitative score, the parameters with a strong effect on the safety factor were shear strength (0.380 point), soil thickness (0.225 point), elastic modulus (0.221 point), fine content (0.056 point), hydraulic conductivity (0.053 point), porosity (0.028 point), water content (0.020 point), and saturation (0.017 point). Additionally, the correlations between factors were calculated by Eq. (4) and plotted as GRG in Fig. 20. GRG shows the relationships between variables, and results close to + 1 (proportion) or – 1 (inverse proportion) mean a high correlation. Note that the values in the diagonal direction, which are the results of comparing the same variable, are all + 1, and values based on + 1 to the left and right are the same. The variables with the highest positive correlation are saturation and water content, because they represent the characterization of fluid. Among the positive correlations, the smallest value is 0.27 for shear strength and soil thickness. The closest value to – 1 (inverse proportion) is – 0.54, which is the relationship between saturation and elastic modulus, and the GRG of hydraulic conductivity and elastic modulus is – 0.073, indicating a relatively small relationship. Saturation and water content are highly correlated and thus important, as shown in Fig. 20, with a low score because they can be linked.

## Discussion

**Figure 21:**
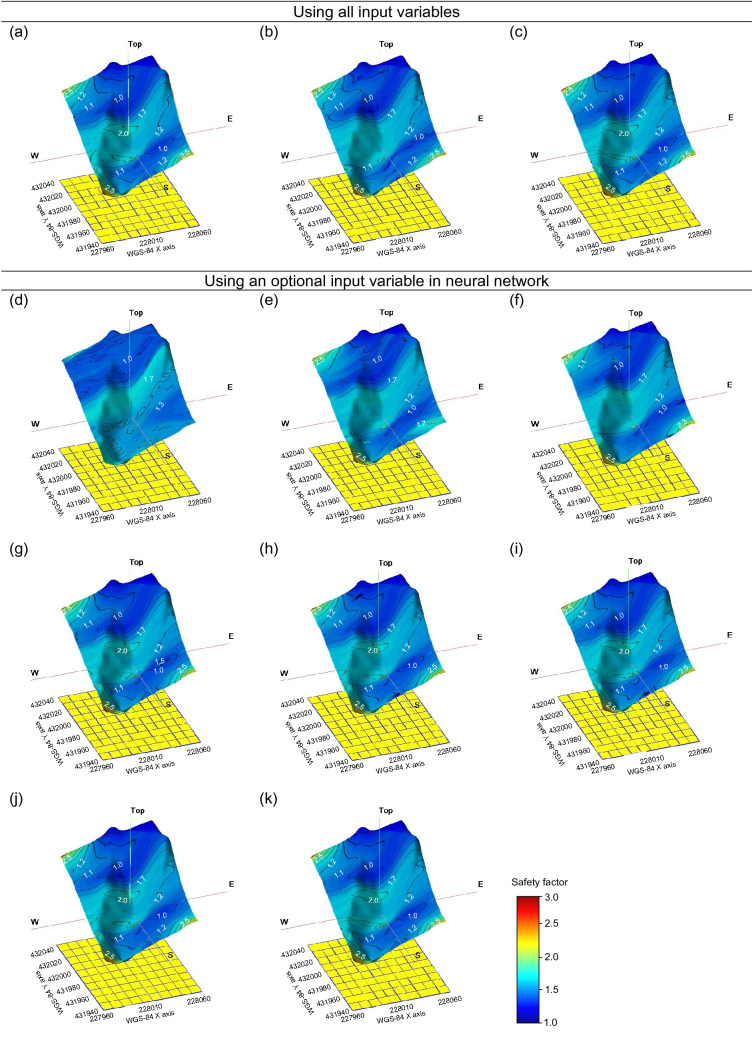
Safety factor modeling based on: (**a**) Eq. (1), (**b**) linear regression; (**c**) neural network; (**d**) 1 parameter; (**e**) 2 parameters; (**f**) 3 parameters; (**g**) 4 parameters; (**h**) 5 parameters; (**i**) 6 parameters; (**j**) 7 parameters; (**k**) 8 parameters. The software of SGeMS beta (v.2.5b) was applied to draw this figure.

Contour maps of the safety factor are plotted in Fig. 21 through LR and NN models with eight variables. Note that the contour map can accurately check the distribution of the safety factor while connecting the same safety factors. In addition, the safety factor calculated by Eq. (1) is shown to be a true value in Fig. 21a. Note that the overall distribution of the safety factor based on LR and NN models is mostly similar to the contour map of the safety factor deduced by Eq. (1). In the linear regression, the distribution of the safety factor at the top and bottom of the slope is more widely spread at a constant value (FS = 1.0), and the color is darker blue. It is also distributed at a constant value (FS = 2.0) in the middle of the slope,thus, the LR method has a limitation in reflecting variations of the safety factor. However, the contour map analyzed by the NN shows similar trends of color and distribution at the upper, middle, and lower slopes with the results based on Eq. (1). In particular, the NN has a characteristic of reflecting various changes in the safety factor in the left and middle areas of the slope. A similar behavior was demonstrated by RMSE and MSE of LR and the NN, which were quantitatively compared with Eq. (1), as shown in Figs. 13 and 16. Therefore, an additional analysis of important input variables based on the NN was attempted due to the relatively high resolution.

Although the deterministic method proposed in this study is also composed of eight variables, it also has a limitation in obtaining all input variables. By adding variables one by one based on the importance results of the RF, the contour maps in Fig. 21 were constructed, in order to find the smallest input variable compared with the resolution through Eq. (1) and to increase the utility of the deterministic method. Soil thickness, elastic modulus, fine content, hydraulic conductivity, porosity, water content, and saturation were added step by step to shear strength based on Fig. 19. The same hyperparameters were applied, referring to the results of Fig. 16. Figure 21k, which is the result of adding all eight input variables, shows the same contour map as Fig. 21c because it is based on the NN with all input variables. Although shear strength and soil thickness are the most important factors in the NN from RF, the contour maps of shear strength and shear strength + soil thickness roughly reflect the accrued value of Fig. 21a. In addition, the constant safety factor at the top and bottom is widely distributed with slight differences from the actual values. However, Fig. 21f, which is the result of applying three important factors (shear strength + soil thickness + elastic modulus) as input variables, shows a similar distribution of the safety factor with the contour map of Fig. 21a. It can be seen that the areas in which the safety factor of the upper left and lower right parts is 2.5 are also reflected. When four important factors (shear strength + soil thickness + elastic modulus + fine content) are selected as input values, the resolution is more similar to Fig. 21a, and the contour map from five input variables shows even more similarity, as shown in Fig. 21h–k.

**Figure 22:**
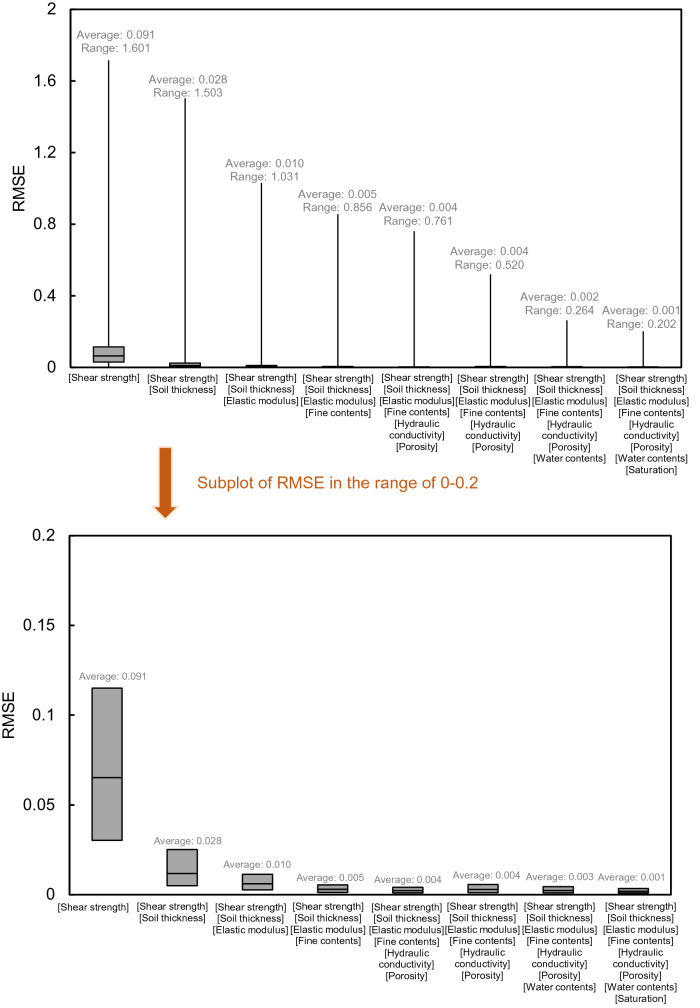
Box plot of RMSE according to the optional input variable in the neural network.

RMSE using one to eight important factors as input variables is shown in Fig. 22 to quantitatively compare the difference in the safety factor according to the important factors. The average RMSE based on one important factor of shear strength was calculated as 0.091, and when all eight variables were entered, the average RMSE was calculated as 0.001, which is 98% less. The more important factors added, the lower the RMSE. A similar range of average RMSE was estimated to be about 0.003–0.005 when four important factors (shear strength + soil thickness + elastic modulus + fine content) and seven important factors (shear strength + soil thickness + elastic modulus + fine content + hydraulic conductivity + porosity + water content) were applied. These results suggest that, if it is difficult to obtain all eight input variables when using an NN, a reliable contour map can be derived by capturing the order of important factors. In addition, it is judged that the assessment of quantitative susceptibility is possible by using at least four important factors: shear strength, soil thickness, elastic modulus, and fine content.

**Figure 23:**
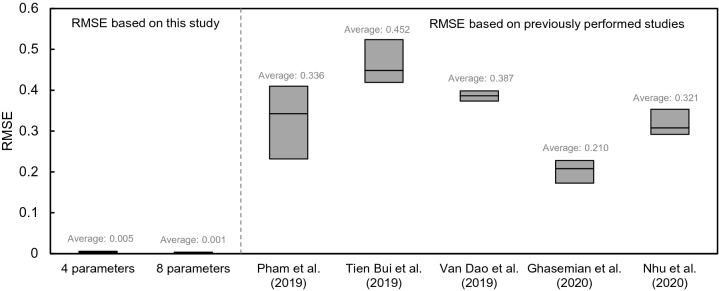
Comparison of RMSE based on this study and previously performed studies.

The RMSE was also used to verify results of this study and the comparison of RMSE was demonstrated in Fig. 23 with previously studied values. To reliably compare RMSE, previous studies that recently performed to estimate landslide susceptibility through machine learning algorithm were selected. And thus, studies conducted by Pham et al.^65^, Tien Bui et al.^17^, Van Dao et al.^14^, Ghasemian et al.^18^ and Nhu et al.^66^ were selected because they used to ensemble technique, novel hybrid function, spatially explicit deep learning neural network, reduced error pruning tree, and combinated ensemble model, respectively for mapping risk area of landslide. The average value of RMSE shown in each paper is 0.336, 0.452, 0.387, 0.210 and 0.321 for Pham et al.^65^, Tien Bui et al.^17^, Van Dao et al.^14^, Ghasemian et al.^18^ and Nhu et al.^66^ respectively. However, the RMSE of this study was found to be 0.005 and 0.001 on average, which are relatively low values when four main parameters and eight parameters were used. These results show different ratios of 41–89.4% and 209–451% based on the four main parameters and eight parameters, respectively. Even though the input variable and selected algorithms were different between this study and the previous studies, the comparison of RMSE show that the methodology used in the study is reliable when evaluating landslide susceptibility through machine learning. The relatively low RMSE is dominated by two factors: 1) input data and 2) machine learning algorithm with hyperparameter. It shows that the eight main variables in empirical equation, constructed by the opinions of expert group, excellently reflect landslide susceptibility considering characterizations of solid, structural failure, and water flow, and high quality data was used as input variables through various experiments. In addition, optimally selected machine learning algorithms and hyperparameter produced reliability result as shown in Figs. 12 and 15. Even though the accuracy depends on geological condition, the number of data and characterization of soil, the applied method in this study is a wise option to estimate landslide susceptibility.

It is difficult to find 8 variables through laboratory and field tests. However, the elastic wave survey is a prior investigation method to understand the entire range of the mountain, and the elastic modulus, which is one of the four main factors, can be obtained through an elastic wave survey. It can also provide the location of the DCPT to obtain soil thickness and shear strength. Finally, fine content is also captured after sampling at the same location with the penetration test. In this way, all four main factors can be obtained. Of course, obtaining hydraulic conductivity, porosity, saturation, and water content, if possible, is also necessary to improve reliability.

## Conclusion

In this study, the weight of each variable in the empirical equation was estimated using LR and NN, and the distribution of the safety factor was addressed. The detailed conclusions are as follows:

- Field and laboratory experiments were conducted to obtain eight variables (fine content, soil thickness, porosity, elastic modulus, shear strength, hydraulic conductivity, saturation, and water content), and the dataset was built through the interpolation method.
- The gradient descent, Levenberg–Marquardt (LM), and Bayesian regularization (BR) methods were used to more precisely perform back propagation, and LM and BR showed excellent performance.
- The safety factor estimated by the NN was most similar to the true value, and the safety factor calculated by the four main factors of shear strength, soil thickness, elastic modulus, and fine content also showed high reliability. Although it is difficult to obtain all eight variables used in the study, the quantitative susceptibility of slope can be evaluated by using only these four main factors.
